# Invited discussant comments during the UCL–Penn Global COVID Study webinar ‘Reflections, Resilience, and Recovery: A qualitative study of Covid-19’s impact on an international adult population’s mental health and priorities for support’: part 2 of 3

**DOI:** 10.14324/111.444/ucloe.100006

**Published:** 2022-12-01

**Authors:** Morgan Vine

**Affiliations:** Head of Policy and Influencing at Independent Age, West Kensington, London, UK

**Keywords:** psychological, people, support, Covid-19, older, old age, ageing, benefits, pensions, poverty, pandemic, loneliness, mental health, physical health, health, wellbeing

## Abstract

This discussant commentary considers the findings presented from the UCL–Penn Global COVID Study webinar ‘Let’s Talk! What do you need to recover from Covid-19?’. The research presented highlights a number of key issues that have affected people of all ages throughout the pandemic. Our aim with this article is to reflect on these themes and, using our own qualitative and quantitative research conducted throughout the pandemic, explore whether the people we spoke to in later life expressed challenges, concerns and frustrations with the same issues as those expressed in Dr Wong’s study. As a national charity that supports people in later life, Independent Age has been incredibly concerned by the impact of the pandemic specifically on people aged 65 and over, and believe more must be done by decision-makers in the government and National Health Service (NHS) to support them to recover from the pandemic.

**About the study**

The UCL–Penn Global Covid Study, launched in April 2020, is a 12-month longitudinal study of the impact of Covid-19 on social trust, mental health and physical health. The Covid-19 global pandemic can be seen as a ‘natural stressor’ or major change in the environment that allowed researchers to study how changes in the environment can have an impact on individuals’ relationships with others and their health. In collaboration with six institutions from Italy, Singapore, the USA, China and the UK [1], the study looks at the short- and longer-term effects of Covid-19 on individuals’ mental health and social relationships with others. Survey data was collected at three timepoints: 17 April to 14 July 2020 (wave 1), 17 October 2020 to 31 January 2021 (wave 2), and 17 April to 31 July 2021 (wave 3).

**About the webinar**

Held online between 2 June and 28 July 2021, the study group presented study data at five online webinars as part of the UCL Global Engagement Fund sponsorship, to discuss the lessons learned. Policymakers and other subject experts were invited to speak on the policy relevance and implications of the study findings. A conscious decision was made by the study team to situate the study findings of individuals’ health and relationships in the context in which they occur – local communities and countries. The recorded comments from these discussions, focusing on the policy relevance and implications of each academic article, were recorded as discussant articles and published in this journal to be read alongside the research article being discussed.

These discussant articles are reviewed by members of the Editorial Board before being published. It is hoped that these discussant articles, read alongside the research articles, will provide a more holistic understanding of the issues at hand, how findings may inform policies in the coming months and/or assist in future crisis management strategies and aid decision-making, in an open and transparent manner.

The study was pre-registered (https://osf.io/4nj3g/ on 17 May 2021) and ethical approval was obtained from the IOE (Institute of Education), UCL’s Faculty of Education and Society (University College London, UK) Ethics and Review Committee on 8 April 2020 (REC 1331) [2].

**Linked research article**

The linked research article to this discussion article cited here has been published in *UCL Open: Environment* following open peer review and made freely available to read as an open access article. Additionally, all previous versions and peer review reports are freely available to read as open access preprint articles from the journal’s preprint server by following the below DOI link and navigating to the version history of the published research article. Readers can find more information about how peer review works in the journal at ucl.scienceopen.com.

Wong KK, Loke K, Melville KMK. Reflections, Resilience, and Recovery: A qualitative study of Covid-19’s impact on an international adult population’s mental health and priorities for support. *UCL Open: Environment*. 2022;(4):12. DOI: https://doi.org/10.14324/111.444/ucloe.000041.

**Recorded webinar**

This discussion article comments on the findings of the research article presented during the following webinar that has been recorded and made freely available to readers to watch on-demand.

Summer Webinar 5 – Let’s Talk! What do you need to recover from Covid-19? #GlobalCOVIDStudy. Available from: https://www.youtube.com/watch?v=i8z9KzIIcj0.

## Introduction

The UCL–Penn Global COVID Study explores important themes when it comes to the recovery of people of all ages from the pandemic. At Independent Age, we want to highlight the specific needs of people in later life. We believe it is essential that older people do not miss out on the support they need to thrive and recover from the impacts of Covid-19. Not because they are more important than younger generations, but because they are as important. This is not the time to stoke the flames of intergenerational tension which we have seen too often through the pandemic, rather to build a recovery plan for us all.

## Discussant comments

Independent Age is a national charity [3]. Our mission is to ensure that, as we grow older, we all have the opportunity to live well; with dignity, choice and purpose. To help achieve this, we do three core things:

- We deliver information, advice and connection services for people aged 65 and over and their families.
- We share best practice, facilitate and fund capacity building in our sector.
- We campaign for positive change on the issues that older people tell us matter most.

Many of the findings included in the UCL–Penn Global COVID Study chimed with the qualitative and quantitative research we have conducted, looking at what people aged 65 and over need to thrive and recover from the pandemic. Since the Covid-19 pandemic began, we have conducted four surveys, the most recent in June 2021. Across these we heard from thousands of older people and learned how their situation was changing.

One limitation to note of our own research was that a large proportion of the people we connected with were online. Our loneliness survey did access some people who were offline, but it was challenging connecting with this group. However, we believe it is a rational assumption that, for those who are digitally disconnected, the problems we highlight could be even worse.

Our research has much in common with the UCL–Penn Global COVID Study, with key themes emerging around access to mental health services, and the importance of personal support and understanding from others. We were interested to see the findings in the UCL–Penn Global COVID Study on the need for financial support from employers and government incentives. When it comes to the people we support, who are aged 65 and over, many are in or near retirement so employment support did not come through in our own research. However, financial security and money worries came up strongly as a significant concern for many.

### Financial security

There are now over two million people aged 65 and over living in poverty in the UK and our own analysis at Independent Age shows this number has risen 5% since 2011 [4].

In autumn 2020, out of 5000 older people who responded to our Home Truths survey [5], almost one in four (24%) said they were feeling more financially insecure because of the pandemic. The most common responses were people who said they faced extra costs for day-to-day living, those who had lost income, and people who were worried about the potential impact of a recession and the state of the economy.

With over one million people aged 65 and over instructed to shield, and everyone aged over 70 told they were clinically vulnerable and at more risk from the virus, people in later life found themselves spending more time at home to protect themselves, their loved ones and the NHS from the virus. This had financial consequences for many. People shared with us the additional costs of meeting minimum spends and delivery charges when online shopping, and they spoke about higher prices when shopping at smaller local stores when trying to avoid public transport. One respondent told us ‘the cost of living has increased. Most times I have to take items off my grocery list because I cannot afford them’. Others shared their worries around rising energy bills, even during the spring and summer when costs are normally lower. This is extremely concerning as many people in later life are already vulnerable to fuel poverty.

Our findings echo those from the UCL–Penn Global COVID Study where participants spoke about financial support being needed during the recovery phase for vulnerable groups including people in later life, with many participants voicing the need for ‘funding for any debt that has occurred because of the pandemic’.

At Independent Age, we want to ensure that older people are not having to make decisions between eating and heating their home. To make this a reality, people in later life living on a low income need to know about, and be able to access, the benefits they are entitled to; receive advice to maximise their savings and pension income; and ensure they are on the most efficient and cost-effective fuel tariffs.

At the moment this is not the case across the board.

That is why we have been running our Credit where it’s due campaign, calling on the government to ensure that everyone entitled to the state benefit pension credit receives it [6]. Currently, one million people are missing out and around £2 billion each year does not reach those who are eligible for it [7].

### Personal support

We support the findings in the UCL–Penn Global COVID Study where participants stated that the presence of a close support system would be important for coping, including more human interactions and the ‘opportunity to visit family’. This also came through strongly in multiple strands of our research.

During the early stages of the pandemic we co-chaired a task force supported by the Department for Digital, Culture, Media and Sport (DCMS). As part of this we undertook new research about loneliness and social connection. Six hundred individuals responded to our survey by phone and online [8,9]:

- 74% said they lacked companionship and felt left out some of the time, or often.
- 82% said they felt isolated from others some of the time, or often.
- Almost three in four (74%) said they felt lonely at the time of the survey, and 9% said they always felt lonely.

When asked how the pandemic had affected them:

- 72% of respondents said their contact with organisations that they used to interact with before the pandemic had decreased.
- 73% said that the Covid-19 pandemic had made them feel significantly or somewhat more lonely or isolated than they did before.
- However, one in four (23%) said they felt the same levels of loneliness as before the pandemic.

The task force concluded that whilst loneliness was new for some, many people in later life were already experiencing high levels of loneliness and isolation prior to the pandemic and, for a significant proportion, that had got worse. This is backed up by our Independent Age advisers, who have confirmed their calls have gotten much harder with callers incredibly anxious and lonely.

Anyone can feel lonely and unsupported, but this lack of personal support and connection will be felt even more severely by those grieving through Covid-19.

Death has been a major part of Covid-19, with people of all ages experiencing unexpected grief. At Independent Age, we estimate that more than 300,000 people aged 65 and over were bereaved of a partner within the first year of the pandemic up until March 2021 (due to Covid and other factors). The death of a partner is a significant life event at any time and one that will have a huge impact on the majority of people in this situation but, during Covid-19 we have heard from people across the country about their specific struggles with grief, in part because they have been unable to access support from their friends and family [10]. One survey respondent shared: ‘I lost a close friend and was not able to attend the funeral or go and see her family, which has made it an unreal event and I felt sad and disassociated at the same time’. This echoes the powerful findings in the UCL–Penn Global COVID Study about the importance of personal connections, and the support these can offer in times of crisis.

Bereavement support can take many forms, both formal and informal, but the research we have undertaken shows that is not readily available in many local areas with bereavement services not being consistently commissioned [11]. At Independent Age, we believe it is vital that the government and the health service take responsibility for this important issue and invest in the bereavement support services which people tell us they need – whether that is counselling, group therapy or mental health support.

### Challenges accessing mental health support

A significant number of people who have been bereaved during Covid-19 could be at risk of experiencing complicated or prolonged grief, due to the restrictions that have been in place during the pandemic. Professional mental health support is often needed to support someone through complicated grief, and it can also help people to deal with low mood, anxiety and depression caused by a range of other factors, not just bereavement.

We note that in the UCL–Penn Global COVID Study, access to mental health came out strongly, with a particular focus on counselling needing to be provided for children. In our view, people of all ages have struggled significantly throughout the pandemic, and many of the worries and concerns expressed by the younger generation will be mirrored by the older. It is therefore imperative that people in later life are not overlooked when it comes to accessing this vital support.

Through our Independent Age research, 66% of the 5000 respondents to our survey in autumn 2020 told us they felt worried or anxious about the impact Covid-19 could have on their life. In addition, almost half (42%) of respondents reported that their mental health had become worse or much worse since the start of the pandemic. When you consider these respondents were all online, we are concerned that the impact on mental health for those offline could be even more severe.

In our Minds that Matter research [12], released in October 2020, many interviewees shared that talking therapy was a big help when trying to improve their mental health. However, concerningly, not enough people in later life are told about, or offered, this treatment. Before the pandemic people aged 65 and over made up only 6% of referrals to NHS England’s talking therapy programme, and during the pandemic this statistic has dropped to 5%. At Independent Age, we strongly believe that as part of the recovery it is vital that more people in later life have access to this important treatment.

### Physical health problems

Physical health problems can negatively impact people’s mental health, and every other aspect of their life. This came out as a top priority in our research. We are surprised that this theme did not surface in the UCL–Penn Global COVID Study.

People told us that Covid-19 had significantly affected their physical health, regardless of whether or not they contracted the virus. With such a large proportion of over 65s told to shield, and many more feeling too worried to leave their homes, the mobility of people in later life has been severely affected, with some telling us they have gone from being able to go for walks, to being housebound and unable to easily get off their sofa. One survey respondent shared: ‘My mobility has got worse as my knee replacement operation was postponed and my shoulder problems have become more painful. My GP was unhelpful about this and my decreasing mobility, especially going up and down stairs, worries me as I live alone’.

Nearly 50% of our survey respondents had experienced some type of problem with their treatment; from accessing medication, to being able to book GP or specialist appointments. 16% told us that their regular healthcare or treatment had been postponed or cancelled because of the pandemic. This is obviously concerning as diagnosis and treatment for serious issues will affect people’s ability to recover from them. One respondent shared: ‘I can’t see consultants. I received a severe lack of communication and the overwhelming impression is that I don’t matter because I’m old’.

We believe that there are both big and small changes needed which would significantly improve people’s experience of accessing treatment to ensure they can recover and thrive after the pandemic. For example, communicating effectively with people while they are on the waiting list, and offering information about exercises and activities that they could do to stay as healthy as possible before their operation.

## Conclusion

In conclusion, our research and regular contact with people in later life supports the findings presented by the UCL–Penn Global COVID Study about the priorities needed to recover and thrive after the Covid-19 pandemic. However, we believe there is an additional priority of ensuring people can access the treatment they need for physical health problems.

Whilst the UCL–Penn Global COVID Study highlights that many of the issues discussed impact people of all ages, for older people their combination of issues can be more difficult to overcome and detract from their ability to enjoy a happy, connected and purposeful later life – which is, after all, what we all aspire to.
